# Differential Identification of Mycobacterial Species Using High-Resolution Melting Analysis

**DOI:** 10.3389/fmicb.2017.02045

**Published:** 2017-10-23

**Authors:** Azar D. Khosravi, Mohammad Hashemzadeh, Abdolrazagh Hashemi Shahraki, Ali Teimoori

**Affiliations:** ^1^Infectious and Tropical Diseases Research Center, Health Research Institute, Ahvaz Jundishapur University of Medical Sciences, Ahvaz, Iran; ^2^Department of Microbiology, School of Medicine, Ahvaz Jundishapur University of Medical Sciences, Ahvaz, Iran; ^3^Department of Epidemiology, Pasteur Institute of Iran, Tehran, Iran

**Keywords:** non-tuberculous mycobacteria, HRM assay, PCR, *rpoBC* locus, melting curve

## Abstract

Infections caused by non-tuberculous mycobacteria (NTM) is increasing wordwide. Due to the difference in treatment of NTM infections and tuberculosis, rapid species identification of mycobacterial clinical isolates is necessary for the effective management of mycobacterial diseases treatment and their control strategy. In this study, a cost-effective technique, real-time PCR coupled with high-resolution melting (HRM) analysis, was developed for the differentiation of Mycobacterial species using a novel *rpoBC* sequence. A total of 107 mycobacterial isolates (nine references and 98 clinical isolates) were subjected to differentiation using *rpoBC* locus sequence in a real-time PCR-HRM assay scheme. From 98 Mycobacterium clinical isolates, 88 species (89.7%), were identified at the species level by *rpoBC* locus sequence analysis as a gold standard method. *M. simiae* was the most frequently encountered species (41 isolates), followed by *M. fortuitum* (20 isolates), *M. tuberculosis* (15 isolates), *M. kansassi* (10 isolates), *M. abscessus* group (5 isolates), *M. avium* (5 isolates), and *M. chelonae* and *M. intracellulare* one isolate each. The HRM analysis generated six unique specific groups representing *M. tuberculosis* complex, *M. kansasii*, *M. simiae*, *M. fortuitum*, *M. abscessus*–*M. chelonae* group, and *M. avium* complex. In conclusion, this study showed that the *rpoBC*-based real-time PCR followed by HRM analysis could differentiate the majority of mycobacterial species that are commonly encountered in clinical specimens.

## Introduction

The genus Mycobacterium encompass several acid-fast bacilli (AFB), including *Mycobacterium tuberculosis* complex, *Mycobacterium leprae*, and non-tuberculous mycobacteria (NTM) (Bottai et al., 2014). The number of NTM species is increasing dramatically with the number of more than 190 species and subspecies in 2017^1^, mainly due to the progresses in identification techniques (Tortoli, 2014). Despite the fact that NTM are typically environmental organisms, several species have been known to be important human pathogens, and recently their infections have been increasingly reported (Johnson and Odell, 2014). According to the American Thoracic Society (ATS) guideline, clinically isolated NTM should be identified to the species level to determine their clinical significance, infection control, epidemiological analysis, and patient management (Griffith et al., 2007). Traditional methods including the phenotypic tests are slow, cumbersome and often not definitive (Kent and Kubica, 1985; Springer et al., 1996; Tortoli, 2003). The PCR-based sophisticated techniques such as sequencing, PCR-restriction fragment length polymorphism analysis (PRA) and microarray analysis are still time-consuming, need cumbersome process and occasionally lead to inaccurate species identification (Lim et al., 2008; Li et al., 2009; Wang et al., 2010; Dai et al., 2011). Several multiplex real time PCR have been used for rapid identification of mycobacterial species but species identification is restricted to the number of species-specific primers (Tobler et al., 2006; Richardson et al., 2009; Ngan et al., 2011; Reddington et al., 2011). High-resolution melting curve (HRM) analysis is a homogeneous, closed-tube post-PCR method for identifying single nucleotide polymorphisms (SNPs), novel mutations, methylation patterns, and species identification. Recently, HRM assay has been used as a simple, low cost and rapid method in mycobacterial research works such as investigation of drug resistance among *M. tuberculosis* (Pietzka et al., 2009; Ramirez et al., 2010), or mycobacterial species identification (Perng et al., 2012; Issa et al., 2014; Chen et al., 2017). However, most of the latter analyses could only discriminate NTM into group or complex level. In this study, we developed a rapid real-time PCR- HRM assay targeting *rpoBC* locus. This target is used for the first time for species identification of mycobacteria.

## Materials and Methods

### Sample Collection and Bacterial Strains

In this experimental study, eight reference strains including laboratory standard strains (*M. tuberculosis* H37Rv ATCC 27294, *M. bovis* BCG Pasteur ATCC 35734, *M. abscessus* ATCC 19977, *M. chelonae* ATCC 35752, *M. fortuitum* ATCC 49403, *M. kansasii* DSM 44162, *M. simiae* ATCC, *M. avium* ATCC 25291), and 98 clinical isolates suspected to NTM were tested. Clinical isolates were recovered from the pulmonary specimens at the selected Regional TB Reference laboratories of Iran, from January 2014 to May 2016. The study was approved by Institutional Ethics and Review Board (Code: IR.AJUMS.REC.1395.223), after submission of preliminary proposal and necessary permission for sample collection was granted. All isolates were recovered from clinical specimens containing acid fast bacilli on direct smear. For initial identification, conventional phenotypic and biochemical tests such as growth at 25, 37, and 42°C, pigment production, semi-quantitative catalase test, Tween 80 hydrolysis, arylsulfatase test (3 and 14 days), heat-stable catalase (pH 7, 68°C), pyrazinamidase (4 and 7 days), urease, nitrate reduction test and colony morphology were performed (Mahon et al., 2014). The contaminated samples or the isolates not matching with the ATS criteria for definition of NTM disease were excluded from the study.

### DNA Extraction

The DNA was extracted using the QIAamp DNA Mini Kit (QIAGEN, Germany). In brief, colonies grown on Lowen-Stein Jenson (LJ) medium were harvested and re-suspended in 0.5 ml of sterile double-distilled water and inactivated at 80°C for 20 min. After thermal inactivation, 5 μl lysozyme (10 mg/ml in 10 mM Tris-HCl, pH 8.0) was added and incubated for 15 min at 37°C. Other steps of extraction were performed according to manufacturer’s instructions. DNA concentrations and purity were determined using Nano Drop one (Thermo Scientific NanoDrop, United States) at 260 nm. Purified DNA was stored at -70°C for subsequent experiments.

### Nucleotide Sequencing

For definitive identification, nearly a 500-bp fragment of the *rpoBC* locus was amplified using a set of primers of *rpoB*CF1 (5′-GAGATGGAGTGCTGGGCCATGC-3′) and *rpoB*CR1 (5′-CCGAAGATCTTCTCGCAGAACAG-3′) as previously described (Dai et al., 2011). The cycling condition was 95°C for 1 min, followed by 30 cycles of 95°C for 30 s, 55°C for 30 s, 72°C for 30 s with a final 72°C for 5 min. The amplified PCR products of *rpoBC* locus for each isolate were purified with the Gene JET^TM^ Gel Extraction Kit (Fermentas, Lithuania) as per manufacturer’s instructions. The sequences of the products were determined using an ABI PRISM 7500 Sequence Detection System (Applied Biosystems, Foster City, CA, United States) according to the standard protocol of the supplier.

### Sequence Data and Phylogenetic Analysis

The sequences of *rpoBC* locus for each isolate were confirmed by BLAST separately, and multiple sequence alignment (MSA) were done for our sequences and all existing relevant sequences of mycobacteria recovered from GenBank database, using MEGA6 program (Tamura et al., 2013). Percentages of similarity between sequences of *rpoBC* locus were determined by comparing sequences to an in-house database of *rpoBC* sequences. Phylogenetic trees were obtained from DNA sequences based on 500 bp fragments using the Neighbor-Joining (NJ) method and Kimura’s two parameter (K2P) distance correction model with 1000 bootstrap replications supported by the MEGA6 software.

### Primer Design for Real-Time PCR-HRM Assay

**FIGURE 1:**
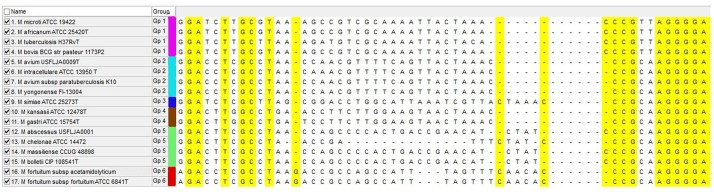
Multiple sequence alignment of *rpoBC* locus for mycobacterial species that are frequently present in clinical specimens. Yellow sequences show conserved region that are suitable for primer designing and central region show hyper variable sequences (intergenic region) that are variable between species. Each HRM group is marked with separate color.

According to the MSA result, we found a hypervariable region flanked by conserved area, suitable for genus specific universal primer designing. Based on this suitable region, a forward primer (5′-AAT CAA CCT GTC GCG CAA CGA-3′) and a reverse primer (5′-GTT CAT CGA AGA AGT TGA CGT-3′) were designed by using Gene runner 3.05 software. Sequence lengths ranged from 115 bp in the *M. chelonae* species ATCC 35752^T^ to 126 bp in *M. abscessus* ATCC 19977^T^. This length variability was due to the variances in the intergenic region between *rpoB* and *rpoC* genes (**Figure 1**). The specificity of the primers were checked by BLAST against the non-mycobacterial genus in GenBank. Primers were not bonded to any non-mycobacterial genus and had a 100% specificity for the *Mycobacterium* genus.

### Real-Time PCR-HRM Assay

Real-time PCR was performed with a Type-it HRM PCR Kit (QIAGEN, Germany) on a Light Cycler 480 system (Roche Diagnostics, Switzerland). Each PCR analysis contained one primer pair. The amplification was performed using the following conditions: a pre-incubation step at 95°C for 10 min, followed by 45 cycles of denaturation at 95°C for 10 s, annealing at 65°C for 30 s, and extension at 72°C for 10 s followed by the Tm analysis with increasing temperatures from 60 to 95°C in a 0.2°C s^-1^ slope increment for 10 s. The HRM analysis was performed using Gene Scanning Software Version 1.5.0 (Roche Instrument Centre, Switzerland). The clustering of the melting curves was based on the regions of the melting curve corresponding to the pre-melting, melting, and post-melting regions. The sensitivity assay was performed using a 10-fold serial dilution of the *M. avium* DNA template (10^7^ to 10^1^ genome equivalents ∼50 ng to ∼5 fg respectively) and each set of assays was performed in duplicate samples. The specificity of the assay was evaluated on eight standard non-mycobacterial isolates, including *Legionella pneumophila* (ATCC 33153)*, Nocardia farcinica* (ATCC 3318)*, Streptococcus pneumoniae* (ATCC 6303)*, Mycoplasma pneumoniae* (ATCC 15293)*, Bacillus subtilis* (ATCC 6633), *Klebsiella pneumoniae* subsp. *pneumoniae* (ATCC 13883), *Pseudomonas aeruginosa* (ATCC 10145), *Enterobacter aerogenes* (ATCC 13048). Distilled water was used to replace the DNA template for the non-template control (NTC).

### Nucleotide Sequence Accession Numbers

The GenBank accession numbers of some investigated isolates of NTM determined in this work are MF109740-109780 (*M. simiae* isolates), MF109782-MF109786 (*M. abscessus* group isolates), MF109787-MF109790 (*M. avium* complex isolates), MF109791-MF109807 (*M. fortuitum* isolates), MF109808-MF109817 (*M. kansasii* isolates), MF109735 (*M. intracellulare* isolate), MF109738 (*M. thermoresistable* isolate), and MF004241 (*M. chelonae* isolate) for *rpoBC* locus.

## Results

The primers which were specifically designed for this assay, successfully amplified all Mycobacterium species. The specific products of mycobacteria were distinguished at the temperature of 83.0–89.0°C. When the assay applied on a range of non-mycobacterial species, the primers always yielded Cq values above 30 (below the detection threshold, with no detectable band on agarose gel electrophoresis).

**FIGURE 2:**
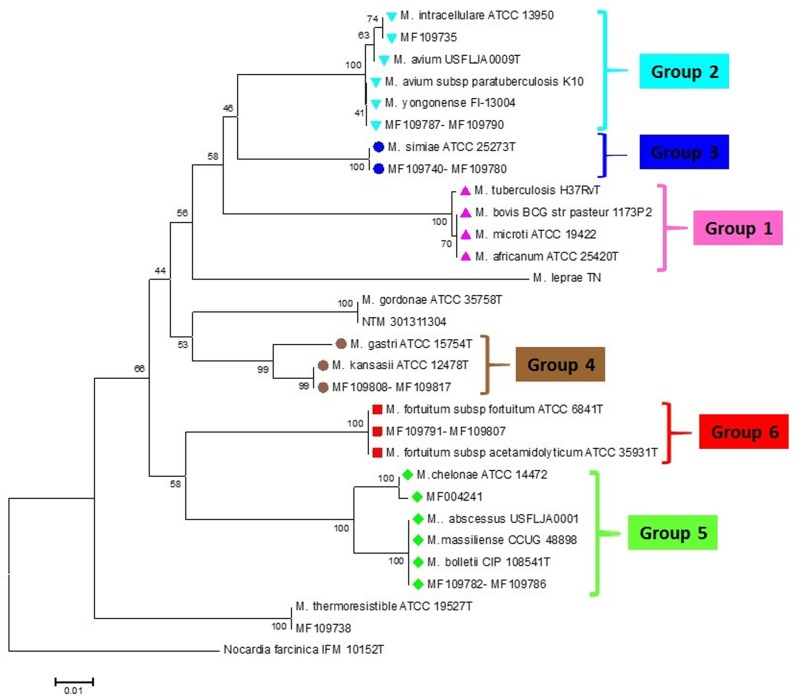
*rpoBC* sequence-based phylogenetic tree of the clinical isolates of NTM with those of closely related species which computed by the NJ analyses and K2P model. The support of each branch, as determined from 1000 bootstrap samples, is indicated by percentages at each node. Bar 0.01 substitutions per nucleotide position. The corresponding HRM groups are specified in the tree.

From 98 mycobacterial clinical isolates identified on the basis of phenotypic and biochemical criteria, 88 (89.7%) isolates were identified at the species level by *rpoBC* locus sequences analysis as a standard method. The neighbor-joining phylogenetic tree based on *rpoBC* sequences of isolates is shown in **Figure 2**. The sequence from *N. farcinica* IFM 10152 was used as an outgroup to construct a rooted tree. The phylogenetic tree based on *rpoBC* was characterized by high robustness within our isolates (almost 80% of the nodes had bootstrap percentages greater than 75%). *M. simiae* was the most frequently encountered (41 isolates), following by *M. fortuitum* (20 isolates), *M. tuberculosis* (15 isolates), and *M. kansassi* (10 isolates). The remaining strains were mostly rare mycobacteria comprising one to five isolates including *M. abscessus* group (5 isolates), *M. avium complex* (5 isolates), and *M. chelonae* and *M. intracellulare* one isolate each. The *rpoBC* locus provided low discrimination within *M. abscessus* subspecies and members of *M. avium* complex, as *M. abscessus* subsp. *abscessus*, *M. abscessus* subsp. *bolletii* and *M. abscessus* subsp. *massiliense* had identical sequences. Also *M. avium* subsp. *paratuberculosis* and *M. yongonense*, belonging to *M. avium* complex, classified in one group.

**FIGURE 3:**
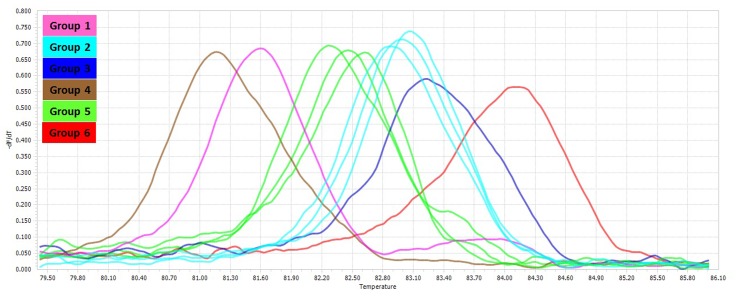
Comparison of melting curves of different non-tuberculosis mycobacteria. Melting curves corresponding to the HRM groups for the mycobacterial *rpoBC* locus.

**FIGURE 4:**
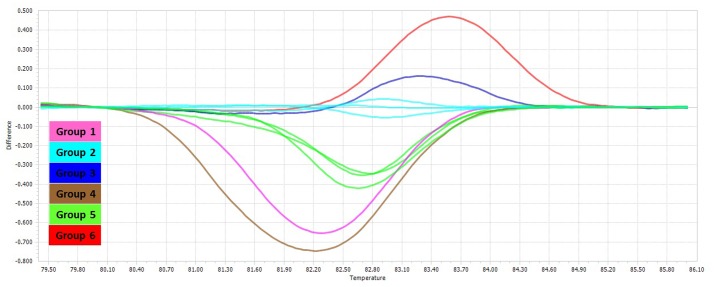
Comparison of HRM different plots of different non-tuberculosis mycobacteria. Difference plot comparison of the six HRM groups for the mycobacterial *rpoBC* locus.

The PCR-HRM assay was performed for differentiation of the mycobacterial species using a designed primer set as shown in **Figure 1**. The analytical sensitivity for HRM assay were conducted on *M. fortuitum* DNAs, assuming that results would be comparable for the other species, since the use of equivalent DNA concentrations yielded almost similar Cq values for all the tested species. The detection threshold was set at 30 cycles, because Cq values of negative controls were always between 30 and 35, probably because of a slight primer–dimer formation that was undetectable by agarose gel electrophoresis and by melting curve analysis. Using this threshold, the assay was able to accurately and reproducibly detect as few as 10 copies of *M. fortuitum*. All 88 clinical strains were classified into six distinct groups. Of these 88 prevalent mycobacterial isolates, 81 mycobacteria (92%) could be identified correctly by real-time PCR-HRM assay. The individual sensitivity and specificity of groups were 90–100 and 97–100% on average respectively. Each HRM groups demonstrated different melting temperature and plot pattern (**Figure 3**). For type categorization, isolates with difference plots within the ±0.2 relative fluorescence unit cut offs, were considered as the “same” type, while isolates with difference plot outside of the ±0.2 RFU cutoffs were denoted as “different.” According to this criteria, *M. tuberculosis* (H37Rv, Ra) and *M. bovis* of the MTBC with 81.60 ± 0.00°C were placed together in group 1. *M. avium*, *M. intracellulare*, and *M. yongonense* of the *M. avium* complex (MAC) (83.00 ± 0.00, 82.90 ± 0.00, and 83.05 ± 0.00°C, respectively), based on the defined cut off, were placed in a single group (group 2). *M. simiae* (83.25 ± 0.00°C), *M. kansasii* (81.15 ± 0.00°C), *M. fortuitum* (84.12 ± 0.00°C) were placed in groups 3, 4, and 6 respectively. *M. chelonae* (82.60 ± 0.00°C) and *M. abscessus* (82.50 ± 0.00°C) with difference plots within 0.2, were categorized together in group 5 (**Figure 4**).

## Discussion

Although sequence based methods are recommended for definitive identification of mycobacterial species (Devulder et al., 2005; Mignard and Flandrois, 2008; Dai et al., 2011; Hashemi-Shahraki et al., 2013; Shahraki et al., 2015), rapid and cost effective techniques such as real time PCR, can be helpful in control and treatment strategies of mycobacterial diseases. In several studies, multiplex real time PCR technique has been used for identification of mycobacterial species (Mokaddas and Ahmad, 2007; Richardson et al., 2009; Ngan et al., 2011; Nasr Esfahani et al., 2012), which a three or four species could be identified in a reaction at maximum. In this study, we developed an in-house PCR-HRM assay targeting *rpoBC* locus, which could successfully differentiate the predominant Iranian clinical mycobacterial species, including *M. tuberculosis*, *M. avium* complex, *M. fortuitum, M. kansasii*, *M. simiae*, and *M. abscessus–M. chelonae* group. Although in theory, HRM assay has high discriminatory power (Yang et al., 2009), in our study, the amplicons of different NTM species (based on *rpoBC* locus sequence) showed the same or similar melting curves. Similarly, the members of *M. tuberculosis* complex, except *M. tuberculosis*, have the same melting curves. Also, members of *M. avium* complex, *M. abscessus*, and *M. chelonae* with different *rpoBC* locus sequence, had difference plots within the ±0.2 relative fluorescence unit cut offs, that could not be categorized them in distinct groups (**Figures 2, 3**). Similar findings have been reported in the literature (Yang et al., 2009; Perng et al., 2012). In this study, of six HRM groups, three (groups 3, 4, 6) were in accordance with the phylogenetic tree obtained from *rpoBC* locus sequences. Perng et al. (2012), evaluated the real time PCR-HRM analysis targeting 16S rRNA gene and ITS region for identification of 134 NTM isolates. Out of 134 isolated isolates, 101 isolates were divided into four groups (*M. avium* complex, *M. chelonae* group, *M. gordonae*, and *M. fortuitum* group). In compare to our results, they could differentiate fewer distinct groups, occasionally with lower sensitivity and specificity. In Malaysia, Issa et al. (2014), developed a qPCR-HRM analysis using 16S rRNA as target gene for the differentiation of the Mycobacterium species. However, they could not identify some of the species that are frequently present in clinical specimens such as *M. simiae, M. fortuitum, M. abscessus*, and *M. kansasii* by their applied method. In a recent study, Chen et al. (2017), high number of clinical NTM species were identified using dual target PCR-HRM analysis. In their study, analysis of the combined 16S rRNA and *hsp65* genes HRM types led to 12 unique HRM patterns, representing 15 different species including *M. avium*, *M. intracellulare*, *M. gordonae*, *M. kansasii*, *M. marinum*, *M. parascrofulaceum*, *M. scrofulaceum*, *M. szulgai*, *M. terrae*, *M. abscessus*, *M. chelonae*, *M. fortuitum*, *M. mucogenicum*, *M. neoaurum, M. smegmatis*. In contrast to our study, high sensitivity and specificity were seen in Chen et al. (2017) study, so that they could differentiate closely related species such as *M. abscessus* and *M. chelonae*. However, we targeted a single locus for species differentiation with the lowest cost.

In current study, the *rpoBC* locus were used for the first time for mycobacterial species identification in a real time PCR-HRM scheme. This assay is reported previously as a robust technique with high discriminatory power (Dai et al., 2011). In contrast, all other similar studies employed *16S rRNA*, *ITS* (Internal Transcribed spacer) or *hsp65* genes as a target gene for PCR-HRM analysis (Douarre et al., 2012; Perng et al., 2012; Phung et al., 2013; Thomson et al., 2013; Issa et al., 2014; Chen et al., 2017).

## Conclusion

Our finding showed that this PCR-HRM assay using *rpoBC* locus as a target, could identify predominant Iranian NTM species in a quick, low-cost and simple method. The total processing time and cost for PCR-HRM assay is significantly much less than PCR-sequencing method. Furthermore, more than 100 samples could be identified simultaneously. Nevertheless, although we investigated prevalent NTM species in clinical samples, it seems that this assay comprise the ability to analyze more NTM species in future studies.

## Author Contributions

AK: substantial contributions to the conception or design of the work; final approval of the version to be published; agreement to be accountable for all aspects of the work in ensuring that questions related to the accuracy or integrity of any part of the work are appropriately investigated and resolved. AHS: substantial contributions to the conception or design of the work; agreement to be accountable for all aspects of the work in ensuring that questions related to the accuracy or integrity of any part of the work are appropriately investigated and resolved. MH: acquisition, analysis, interpretation of data for the work; final approval of the version to be published. AT: analysis, interpretation of data for the work.

## Conflict of Interest Statement

The authors declare that the research was conducted in the absence of any commercial or financial relationships that could be construed as a potential conflict of interest.

## Footnotes

1 http://www.bacterio.cict.fr/m/mycobacterium.html
